# Sex‐specific changes in vital signs and common blood tests on the day of influenza diagnosis

**DOI:** 10.14814/phy2.70486

**Published:** 2025-08-07

**Authors:** Kurt C. Showmaker, Brigitte E. Martin

**Affiliations:** ^1^ Dasomics LLC Madison Mississippi USA; ^2^ Department of Cell and Molecular Biology University of Mississippi Medical Center Jackson Mississippi USA; ^3^ Center for Immunology and Microbial Research (CIMR) Jackson Mississippi USA

**Keywords:** blood tests, biomarkers, influenza, sex differences, vital signs

## Abstract

Early detection of influenza virus infection can reduce morbidity, mortality, and transmission by enabling earlier and more effective interventions. While influenza is primarily a localized respiratory infection, previous studies showed significant changes in pulse rate and temperature on the day of diagnosis. This study expands these findings to further identify diagnostic patterns by analyzing standard blood panels, including complete blood count, metabolic panels, coagulation panels, and C‐reactive protein. We evaluated 1896 influenza‐diagnosed patients using electronic health records, including demographics, vital signs, and blood test results within 60 days before and after diagnosis. We found significant differences in 14 of 19 blood tests on the day of diagnosis. Sex‐specific differences were notable: five tests showed changes in both sexes, while 10 differed by sex. Significant changes for females included increased levels of red blood cells, white blood cells, hemoglobin, glucose, aspartate aminotransferase (AST), and prothrombin time (PT), as well as decreased chloride levels. For males, significant changes included an increased estimated glomerular filtration rate (eGFR) and reduced levels of potassium and creatinine. These findings highlight the diagnostic potential of combining vital signs and blood test differences to distinguish influenza infection and emphasize the importance of considering sex‐specific variations in clinical assessments.

## 1 INTRODUCTION

Influenza‐like illness (ILI) symptoms, defined by the Centers for Disease Control and Mayo Clinic, include fever, chills, myalgia, fatigue, weakness, chest discomfort, cough, runny or stuffy nose, and headaches. Sometimes, vomiting and diarrhea may occur, though these are more common in children than adults (Centers for Disease Control and Prevention, 2022; Mayo Clinic, 2024). However, viral and nonviral pathogens cause these symptoms. Rapid influenza diagnostic testing often yields false negative results, which are influenced by the amount of viral shedding and the timing of testing, ideally within 3–4 days of symptom onset (Centers for Disease Control and Prevention, 2024a, 2024b). Accurate identification of influenza infection is crucial for diagnosing and implementing pharmaceutical and non‐pharmaceutical interventions to reduce morbidity, mortality, and transmission while improving surveillance efforts (Gaitonde et al., 2019; Montgomery et al., 2024; Yang et al., 2015).

Influenza virus transmission is particularly concerning during the presymptomatic phase, as well as in asymptomatic individuals and those with mild or covert symptoms, all of which contribute to the spread of the virus to susceptible populations (Carrat et al., 2008). In addition to strengthening surveillance of circulating viruses, especially in asymptomatic cases, blood test analyses could be a valuable tool in regions with limited access to reagents, specialized laboratories, equipment, and personnel. The timely administration of anti‐influenza drugs, ideally within 48 h of symptom onset or exposure to an infected individual, is critical for reducing viral replication, limiting transmission, and improving outcomes (Centers for Disease Control and Prevention, 2023; Jones et al., 2021).

Recent research has explored blood test analyses as a supplement or alternative to traditional methods for identifying virus‐positive patients, including those with COVID‐19 and influenza (Cunha et al., 2009; Ferrari et al., 2020; Hong et al., 2024; Kukar et al., 2021; Li et al., 2021; Liao et al., 2021; Qi et al., 2024; Shen et al., 2020; Zhou et al., 2021). Nonspecific blood tests, such as C‐reactive protein (CRP) and white blood cell counts (WBC), can help distinguish between viral and bacterial infections. In cases of influenza, a decrease in lymphocyte count is often observed (Jernigan et al., 2011). Combining symptoms with other diagnostic information has also proven an effective strategy (Ebell et al., 2012; Nabeshima et al., 2002). More recently, studies have demonstrated that blood tests alone can enable the classification of approximately 70% of COVID‐19‐positive patients as either positive or negative (Ferrari et al., 2020). Physicians have noted consistent changes in blood parameters among COVID‐19 patients, including hypoalbuminemia and increased CRP (Rodriguez‐Morales et al., 2020).

Distinguishing patterns among standard blood panels, including complete blood count, basic metabolic panel, comprehensive metabolic panel, coagulation panel, and C‐reactive protein, could lead to earlier diagnosis and better prognosis for symptomatic carriers. This approach could also be beneficial in identifying asymptomatic carriers of the virus and enhancing infectious disease surveillance. In this study, we assess vital signs and common blood tests in influenza‐diagnosed patients and compare these measurements to those taken on the day of diagnosis as well as up to 60 days before and after the day of diagnosis.

## 2 MATERIALS AND METHODS

Data extracted from the University of Mississippi Medical Center (UMMC) Patient Cohort Explorer (PCE) (University of Mississippi Medical Center, 2020) contains patient data derived from the electronic health record used on‐site (EPIC) from January 1, 2017, to December 31, 2022. The data warehouse contains de‐identified and date‐shifted clinical information, ensuring it does not include any protected health information (PHI). According to 45 CFR 46, using this database does not qualify as human subjects research and does not require IRB review.

Patients diagnosed with influenza were identified by the encounter diagnosis name “influenza A” or “influenza B,” determined by rapid antigen assay, reverse transcriptase polymerase chain reaction, direct or indirect fluorescent staining, or viral culture. Note that a positive test result defines a diagnosis and may not necessarily reflect a symptomatic state.

For each influenza‐diagnosed patient, we collected all available values for vital signs (diastolic blood pressure, systolic blood pressure, pulse rate, respiratory rate, and temperature) and laboratory parameters (complete blood count, basic metabolic panel, comprehensive metabolic panel, coagulation panel, and C‐reactive protein) from healthcare encounters occurring within ±60 days of the first unique influenza diagnosis. Patients were included regardless of comorbidity status to evaluate population‐level patterns. A detailed summary of the number of influenza‐diagnosed patients and those contributing data within each measurement category, stratified by sex, is provided in Table S1.

This longitudinal study grouped measurements into five time intervals relative to diagnosis: 60–31 days before (−60), 30–1 days before (−30), day of diagnosis (0), 1–30 days after (30), and 31–60 days after (60). Patients could contribute multiple measurements per time bin, resulting in repeated measures per individual. However, due to clinical practice variability, not all patients had data for every variable or time point, resulting in an unbalanced dataset with irregular repeated measures.

Due to the irregular measurement frequency, traditional repeated‐measures analyses were not suitable for this data. Two‐way ANOVA (*α* = 0.05) was used to assess the main effects of time, sex, and their interaction. Post hoc comparisons were conducted using Šídák's multiple comparisons test (*α* = 0.05) to assess sex differences within each time bin and temporal changes within each sex relative to the day of diagnosis. One‐way ANOVA was initially used to explore within‐sex changes across time; however, these results were excluded from the main figures and tables to reduce redundancy. One‐way ANOVA was also conducted to explore within‐sex temporal changes; these results were excluded from main figures and tables to avoid redundancy, as the two‐way ANOVA captured the same patterns through its time and interaction terms.

All statistical analyses and graphing were performed using GraphPad Prism version 10.0.3.

## 3 RESULTS

### 3.1 Patient influenza diagnosis encounters and demographics

There were 19,264 encounters for 16,966 patients identified as influenza‐positive from January 1, 2017, to December 31, 2022. The majority of encounters occurred in 2019, accounting for 26% of the total encounters (Figure S1a). The peak month for influenza diagnoses varied by year: 24% of 2017 encounters were in December, 44% of 2018 encounters were in January, 42% of 2019 encounters were in February, 39% of 2020 encounters were in February, 60% of 2021 encounters were in December, and 43% of 2022 encounters were in November (Figure S1b). Of the 16,966 patients, most had only one influenza diagnosis, with 12% having two or more independent influenza diagnoses (Figure S1c). Most patients (82%) were under 18 years old, with 39% female and 43% male; 48% of patients had a BMI ≤18, with 22% female and 26% male; and 64% of patients self‐identified as Black or African American, with 33% female and 31% male (Table S2).

Of the 1896 influenza‐diagnosed patients with longitudinal data collected, the years with the most encounters were 2017 (22%), 2019 (21%), and 2022 (23%) (Figure S1d). The peak month for influenza diagnoses varied by year: 30% of 2017 encounters were in December, 47% of 2018 encounters were in January, 32% of 2019 encounters were in February, 33% of 2020 encounters were in January and February each, 35% of 2021 encounters were in December, and 48% of 2022 encounters were in November (Figure S1e). For the 1896 patients with measurements, an equal number of patients (50% each) had only one influenza diagnosis, and 50% had two or more independent influenza diagnoses (Figure S1e). Most encounters were classified as “hospital encounters,” encompassing both inpatient and outpatient visits. Encounters before and after influenza diagnosis may represent routine healthcare utilization unrelated to the influenza diagnosis.

### 3.2 Vital signs

**FIGURE 1:**
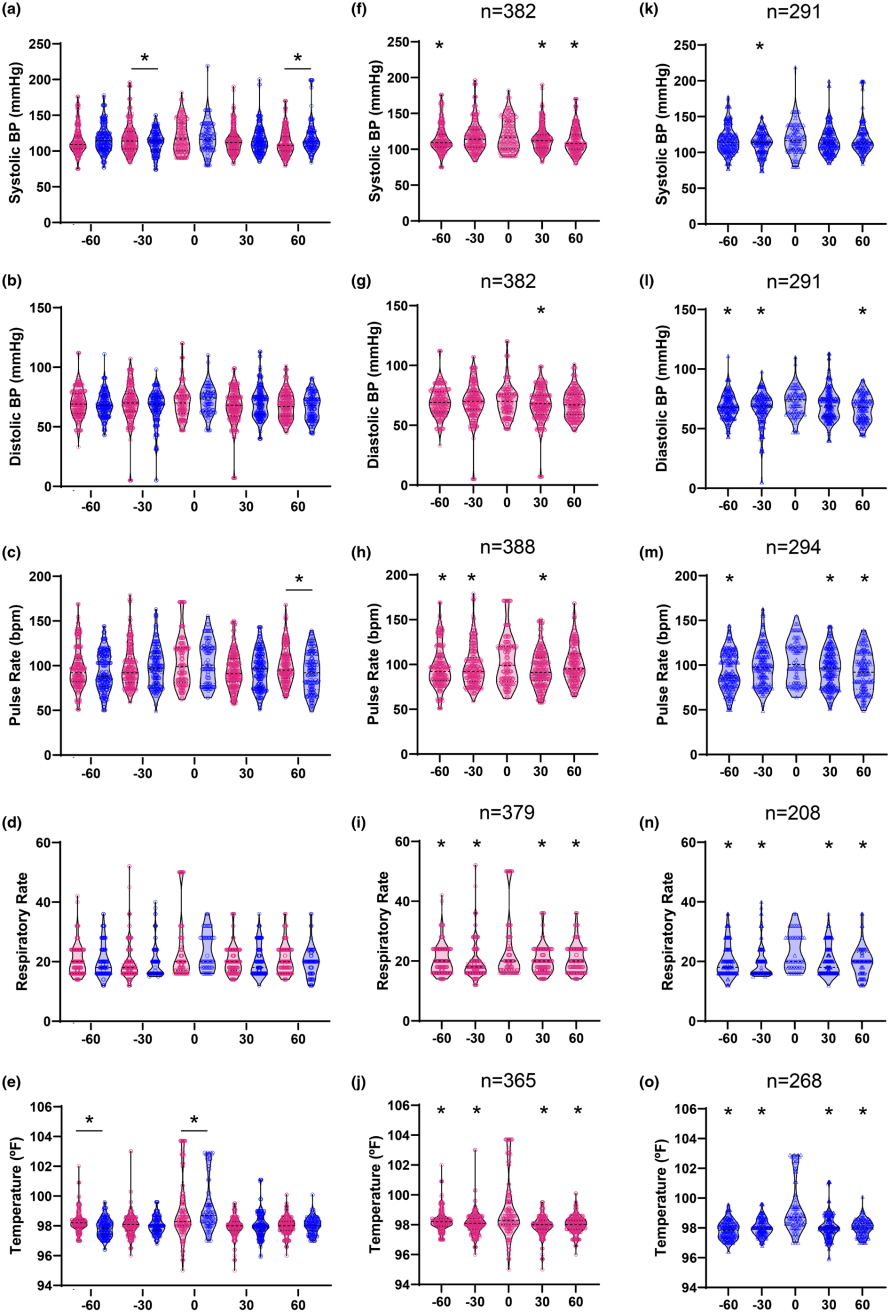
Vital sign dynamics during influenza diagnosis for females (magenta) and males (blue). Each measurement per patient (*n*) was grouped relative to the day of diagnosis − 60 to −31 days before diagnosis (−60), − 30 to −1 days before diagnosis (−30), day of diagnosis (0), 1 to 30 days after diagnosis (30), and 31 to 60 days after diagnosis (60). Dashed lines indicate the mean; dotted lines represent quartiles. Two‐way ANOVA (*α* = 0.05) was used to evaluate the main effects of sex, time, and their interaction. (a–e) between‐sex comparisons at each timepoint using Šídák's post hoc test (*p < 0.05), performed only when a significant main effect or interaction was detected. No post hoc tests were conducted for panel (d) due to the absence of a significant sex effect. (f–o) within‐sex comparisons across time relative to day 0, using Šídák's post hoc test (*p < 0.05).

For all assessed vital signs, there was a statistically significant interaction between sex and time (two‐way ANOVA; *p* < 0.05; Figure 1a–e, Table 1), except for respiratory rate (*p* = 0.9111). On the day of diagnosis, males had a significantly greater increase in temperature than females (1.28°F vs. 0.87°F; *p* = 0.0099). Other differences on the day of diagnosis were not statistically significant. Still, females had greater average increases in systolic blood pressure (5.35 mmHg vs. 2.75 mmHg in males) and respiratory rate (3.43 bpm vs. 3.33 bpm in males). In comparison, males showed larger increases in diastolic blood pressure (3.99 mmHg vs. 1.74 mmHg in females) and pulse rate (9.11 bpm vs. 6.68 bpm in females).

**TABLE 1:** *p* Values for female and male patients relative to their first unique viral diagnosis, grouped into the following time intervals: −60 to −31 days before diagnosis (−60), −30 to −1 days before diagnosis (−30), the day of diagnosis (0), 1–30 days after diagnosis (30), and 31–60 days after diagnosis (60).

	Two‐way ANOVA			Sex					Time							
				Overall Šídák's multiple comparisons test					Females Šídák's multiple comparisons test				Males Šídák's multiple comparisons test			
	Sex	Time	Sex × time	−60	−30	0	30	60	0 vs. −60	0 vs. −30	0 vs. 30	0 vs. 60	0 vs. −60	0 vs. −30	0 vs. 30	0 vs. 60
Vital signs																
Systolic blood pressure (mmHg)	0.2933	**0.0244**	**0.0002**	0.4482	**0.0325**	0.9909	0.9783	**0.0015**	**0.0082**	0.4814	**0.0056**	**<0.0001**	0.8262	**0.0289**	0.2912	>0.9999
Diastolic blood pressure (mmHg)	0.6426	**0.0047**	**0.0076**	0.746	0.0507	0.8744	0.1253	0.9643	0.9503	0.9893	**0.0267**	0.1109	**0.0448**	**0.0026**	0.2421	**0.0037**
Pulse rate (bpm)	0.0566	**<0.0001**	**0.0151**	0.3986	0.9975	>0.9999	0.9938	**0.0016**	**0.0015**	**0.0149**	**<0.0001**	0.1983	**0.0001**	0.126	**0.0022**	**<0.0001**
Respiratory rate (bpm)	0.5392	**<0.0001**	0.9111	–	–	–	–	–	**<0.0001**	**<0.0001**	**<0.0001**	**0.0008**	**0.0011**	**0.0002**	**0.0016**	**0.0014**
Temperature (°F)	0.6194	**<0.0001**	**<0.0001**	**<0.0001**	0.9984	**0.0099**	0.2913	>0.9999	**<0.0001**	**<0.0001**	**<0.0001**	**<0.0001**	**<0.0001**	**<0.0001**	**<0.0001**	**<0.0001**
Complete blood count																
Red blood cells (M/cmm)	**<0.0001**	0.0959	0.4107	**0.0125**	0.7154	0.6295	0.3325	**0.0498**	–	–	–	–	–	–	–	–
White blood cells (TH/cmm)	0.8354	0.1858	0.2305	–	–	–	–	–	–	–	–	–	–	–	–	–
Platelets (TH/cmm)	0.05	**<0.0001**	0.9667	–	–	–	–	–	**<0.0001**	**<0.0001**	**<0.0001**	**<0.0001**	0.6193	0.3644	**0.0005**	**0.0254**
Hemoglobin (g/dL)	0.4903	**0.006**	0.4076	–	–	–	–	–	**<0.0001**	**<0.0001**	**<0.0001**	**<0.0001**	**<0.0001**	**<0.0001**	**<0.0001**	**<0.0001**
Hematocrit (%)	0.5829	**0.0392**	0.4205	–	–	–	–	–	**<0.0001**	**<0.0001**	**<0.0001**	**<0.0001**	0.9957	0.094	0.9075	0.9797
Basic metabolic panel																
Calcium (mg/dL)	0.5945	**<0.0001**	0.9454	–	–	–	–	–	**<0.0001**	**<0.0001**	**<0.0001**	**<0.0001**	**<0.0001**	**0.0001**	**0.0036**	**<0.0001**
Glucose (mg/dL)	0.4621	**0.0439**	0.0559	–	–	–	–	–	**0.008**	0.7792	0.8859	0.3358	0.9894	**0.0451**	0.959	0.9488
Sodium (mmol/L)	**<0.0001**	**<0.0001**	0.2782	**0.0034**	0.2029	0.3967	**0.0295**	0.9784	**<0.0001**	**<0.0001**	**<0.0001**	**<0.0001**	**<0.0001**	**<0.0001**	**<0.0001**	**<0.0001**
Potassium (mmol/L)	**<0.0001**	**0.0121**	**0.0056**	**<0.0001**	**<0.0001**	**0.0055**	**<0.0001**	**0.0139**	**<0.0001**	**<0.0001**	**<0.0001**	**<0.0001**	**<0.0001**	**<0.0001**	**<0.0001**	**<0.0001**
Bicarbonate (mmol/L)	0.0697	**<0.0001**	0.8777	–	–	–	–	–	**<0.0001**	**<0.0001**	**<0.0001**	**<0.0001**	**<0.0001**	**<0.0001**	**<0.0001**	**<0.0001**
Chloride (mmol/L)	**<0.0001**	**0.0422**	0.4374	**0.0025**	**0.0277**	0.4581	0.0945	0.159	**<0.0001**	**<0.0001**	**<0.0001**	**<0.0001**	**<0.0001**	**<0.0001**	**<0.0001**	**<0.0001**
Blood urea nitrogen (BUN) (mg/dL)	**<0.0001**	0.4081	0.4499	**<0.0001**	**<0.0001**	**0.0055**	**0.0004**	**0.001**	–	–	–	–	–	–	–	–
Creatinine (mg/dL)	**<0.0001**	**0.0191**	**0.0207**	**0.0002**	**<0.0001**	0.2225	0.5259	**0.0026**	0.993	0.9054	>0.9999	0.3556	0.1119	**0.0018**	>0.9999	**0.0298**
Estimated glomerular filtration rate (eGFR) (mL/min/1.73 m^2^)	**0.001**	**0.0114**	0.1498	**0.0248**	**0.0422**	0.9872	>0.9999	0.798	0.8386	**0.0307**	0.1225	**0.0114**	**0.0005**	**<0.0001**	0.2428	**0.0018**
Comprehensive metabolic panel																
Albumin (g/dL)	0.6142	0.8465	0.0967	–	–	–	–	–	–	–	–	–	–	–	–	–
Alkaline phosphatase (ALP) (U/L)	0.6812	0.4244	0.094	–	–	–	–	–	–	–	–	–	–	–	–	–
Aspartate aminotransferase (AST) (U/L)	**<0.0001**	**0.0032**	0.7189	0.0975	0.9516	**0.0383**	0.1219	0.6522	**0.0178**	0.0894	**0.0388**	**0.0112**	0.681	0.0966	0.6336	0.1856
Coagulation panel																
Prothrombin time (PT) test (sec)	0.1207	**0.0101**	0.0918	–	–	–	–	–	0.252	0.3753	0.0602	0.9237	0.7285	0.2673	0.8624	0.9006
C‐reactive protein test																
C‐reactive protein (CRP) (mg/dL)	0.1053	**0.0006**	0.7861	–	–	–	–	–	0.0809	0.1382	0.1076	0.0995	0.2839	0.7567	**0.019**	0.1744

*Note*: Two‐way ANOVA (*α* = 0.05) was used to assess the source of variation between sex (female vs. male), time, and the interaction between sex and time; *p* values in columns 2–4. Šídák's multiple comparisons tests (α = 0.05) were applied to (1) compare females and males within each time group (columns labeled “0,” “30,” “60,” etc. under “Overall”), (2) assess within‐sex temporal differences relative to the day of diagnosis (columns labeled “0 vs. −60” through “0 vs. 60” under female and male sections). *p* values <0.05 are in bold. Post hoc comparison was not performed due to nonsignificant main effect in two‐way ANOVA.

For the main effect of time, significant changes were observed across time points for all vital signs, with distinct patterns in each sex (Table 1). Within‐sex post hoc comparisons revealed statistically significant changes on the day of diagnosis relative to other time points in all vital signs for both sexes, highlighting robust physiological shifts associated with influenza diagnosis (Figure 1f–o).

### 3.3 Blood tests

Of the 1896 patients and 19 blood tests, we assessed 12,667 measurements. 367 (19.4%) patients had only one blood test measurement, while the remaining patients had two or more; fewer than 20 patients underwent all 19 blood tests assessed in this study. Glucose and creatinine were the most common measurements, with 1280 (10.1%) and 1020 (8.05%). CRP and prothrombin time were the least common, with only 57 (0.45%) and 116 (0.92%) measurements, respectively.

### 3.4 Complete blood count panel

**FIGURE 2:**
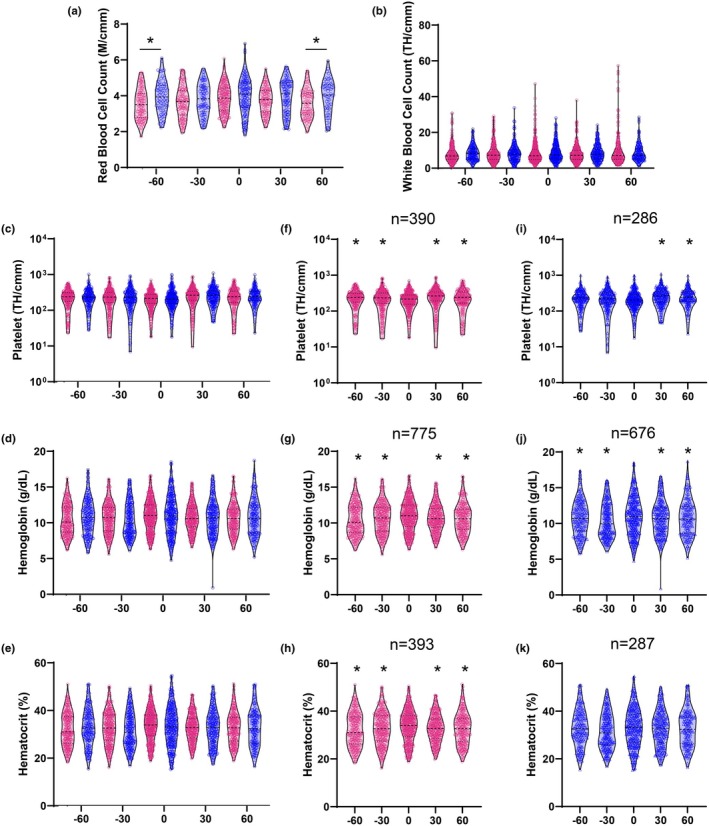
Complete blood count panel during influenza diagnosis for females (magenta) and males (blue). Each measurement per patient (*n*) was grouped relative to the day of diagnosis −60 to −31 days before diagnosis (−60), − 30 to −1 days before diagnosis (−30), day of diagnosis (0), 1 to 30 days after diagnosis (30), and 31 to 60 days after diagnosis (60). Dashed lines indicate the mean; dotted lines represent quartiles. Two‐way ANOVA (*α* = 0.05) was used to evaluate the main effects of sex, time, and their interaction. (a, b) between‐sex comparisons at each timepoint using Šídák's post hoc test (*p < 0.05), performed only when a significant main effect or interaction was detected. (c–e) are included to visualize additional parameters without a significant main effect of sex; post hoc comparisons were not performed. (f–k) within‐sex comparisons across time relative to day 0, also using Šídák's post hoc test (*p < 0.05), shown for parameters with a significant main effect of time.

There was only a significant main effect of sex for red blood cells (*p* < 0.0001; Table 1), with females having higher values than males across all time points. Although not statistically significant between the sexes on the day of diagnosis (*p* < 0.05; Sidak's multiple comparisons tests; Figure 2a–e), female patients had a slightly larger average increase in red blood cells (0.22 M/cmm vs. 0.07 M/cmm for males; *p* = 0.6295) and hematocrit (1.09% vs. 0.63% for males; *p* = 0.8568). Additionally, while females had an average decrease in white blood cells (−0.03 TH/cmm), males had an increase in average white blood cells (0.12 TH/cmm) on the day of diagnosis (*p* = 0.9971). Lastly, males had a larger average increase in hemoglobin (1.3 g/dL vs. 0.84 g/dL for females; *p* > 0.9999) and a larger decrease in platelets (−30.25 TH/cmm vs. −29.8 TH/cmm for females; *p* = 0.8317).

The main effect of time was significant for platelets, hemoglobin, and hematocrit (*p* < 0.05; Table 1). However, a statistically significant interaction was observed between sex and time, indicating similar temporal patterns across sexes. Within‐sex post hoc comparisons revealed significant changes in females for platelets, hemoglobin, and hematocrit on the day of diagnosis (all *p* < 0.0001; Figure 2f–h). In males, platelets and hemoglobin (*p* < 0.05; Figure 2i–k) showed significant temporal changes around the day of diagnosis.

### 3.5 Basic metabolic panel

**FIGURE 3:**
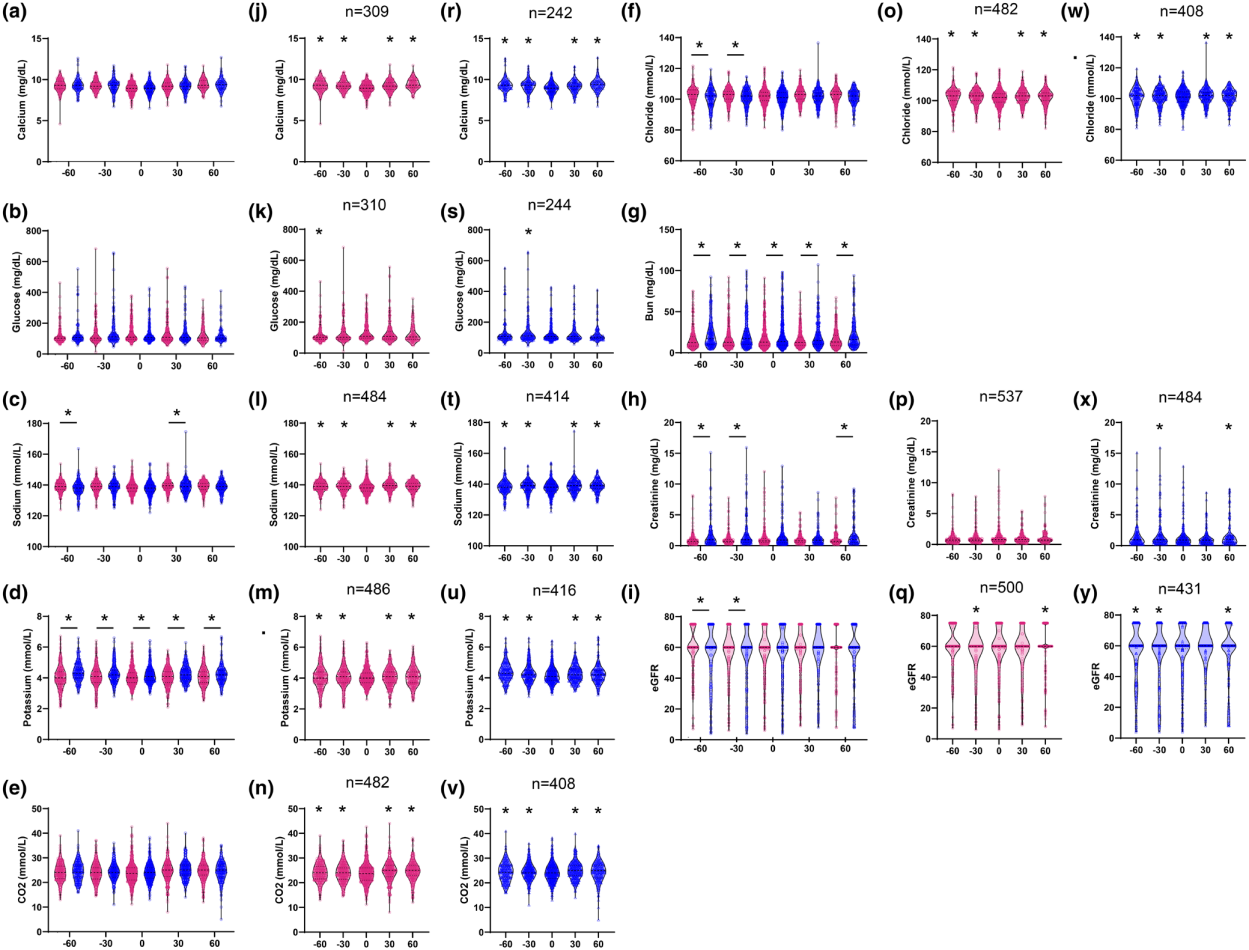
Basic metabolic panel during influenza diagnosis for females (magenta) and males (blue). Each measurement per patient (*n*) was grouped relative to the day of diagnosis −60 to −31 days before diagnosis (−60), −30 to −1 days before diagnosis (−30), day of diagnosis (0), 1 to 30 days after diagnosis (30), and 31 to 60 days after diagnosis (60). Dashed lines indicate the mean; dotted lines represent quartiles. Two‐way ANOVA (*α* = 0.05) was used to evaluate the main effects of sex, time, and their interaction. (c, d, f–i) between‐sex comparisons at each timepoint using Šídák's post hoc test (*p < 0.05), performed only when a significant main effect or interaction was detected. (a, b, e) are included to visualize additional parameters without a significant main effect of sex; post hoc comparisons were not performed. (j–y) within‐sex comparisons across time relative to day 0, also using Šídák's post hoc test (*p < 0.05), shown for parameters with a significant main effect of time.

A significant main effect of sex was observed for sodium, potassium, bicarbonate, chloride, blood urea nitrogen (BUN), creatinine, and estimated glomerular filtration rate (eGFR) (two‐way ANOVA; *p* < 0.05; Table 1). A statistically significant interaction was found between sex and time for potassium and creatinine, indicating that temporal trends differed between females and males for these parameters. On the day of diagnosis, only potassium and BUN showed statistically significant differences between the sexes (Sídák's multiple comparisons test; *p* < 0.05; Figure 3a–i). Males had a slightly larger decrease in potassium compared to females (−0.11 mmol/L vs. –0.10 mmol/L; *p* = 0.0055), while females had an average increase in BUN (1.02 mg/dL) compared to a decrease in males (−0.93 mg/dL; *p* = 0.0055). Although not statistically significant, females had modestly greater decreases in calcium (−0.38 mg/dL vs. –0.33 mg/dL in males; *p* = 0.998), sodium (−1.08 mmol/L vs. –0.80 mmol/L in males; *p* = 0.3967), and chloride (−0.85 mmol/L vs. –0.25 mmol/L in males; *p* = 0.4581), and a greater increase in glucose (8.55 mg/dL vs. –4.62 mg/dL in males; *p* = 0.4141). Males had slightly larger reductions in bicarbonate (−0.57 mmol/L vs. –0.56 mmol/L in females; *p* = 0.8729) and creatinine (−0.36 mg/dL vs. –0.04 mg/dL in females; *p* = 0.2225), as well as greater increases in eGFR (+5.69 mL/min/1.73 m^2^ vs. +2.54 mL/min/1.73 m^2^ in females; *p* = 0.9872).

A significant main effect of time was observed for all parameters except BUN (*p* < 0.05; Table 1). Within‐sex post hoc comparisons revealed significant day‐of‐diagnosis changes for most parameters. In females, significant changes were observed in all parameters except creatinine (Figure 3j–q). In males, all these parameters showed significant changes (Figure 3r–y).

### 3.6 Comprehensive metabolic panel

**FIGURE 4:**
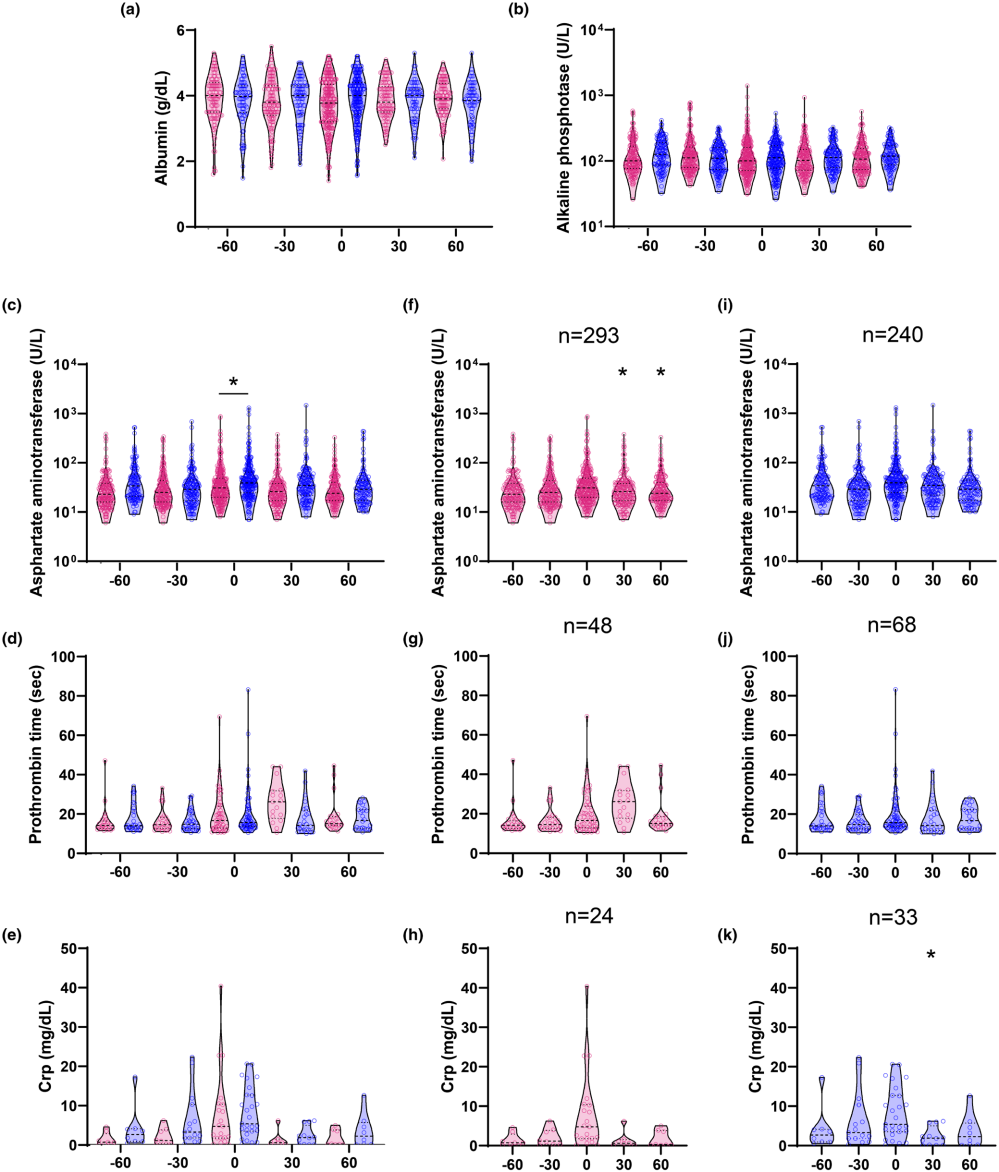
Comprehensive metabolic panel, prothrombin time, and C‐reactive protein (CRP) for females (magenta) and males (blue). Each measurement per patient (*n*) was grouped relative to the day of diagnosis −60 to −31 days before diagnosis (−60), −30 to −1 days before diagnosis (−30), day of diagnosis (0), 1 to 30 days after diagnosis (30), and 31 to 60 days after diagnosis (60). Dashed lines indicate the mean; dotted lines represent quartiles. Two‐way ANOVA (*α* = 0.05) was used to evaluate the main effects of sex, time, and their interaction. (c) between‐sex comparisons at each timepoint using Šídák's post hoc test (*p < 0.05), performed only when a significant main effect or interaction was detected. (a, b, d, e) are included to visualize additional parameters without a significant main effect of sex; post hoc comparisons were not performed. (f–k) within‐sex comparisons across time relative to day 0, also using Šídák's post hoc test (*p < 0.05), shown for parameters with a significant main effect of time.

A significant main effect of sex was observed only for aspartate aminotransferase (AST; two‐way ANOVA, *p* < 0.0001; Table 1), with no significant sex effects for albumin or alkaline phosphatase (ALP; *p* > 0.05). On the day of diagnosis, AST was the only parameter that showed a statistically significant difference between sexes (Sídák's multiple comparisons test; *p* = 0.0383; Figure 4c). Males had a larger average increase in AST (17.26 U/L) compared to females (13.69 U/L). Although not statistically significant, females showed a modest decrease in albumin (−0.14 g/dL), while males had a slight increase (0.06 g/dL; *p* = 0.1205; Figure 4a). Males also had a larger average decrease in ALP (−4.57 U/L vs. –0.6 U/L in females; *p* = 0.9745; Figure 4b).

A significant main effect of time was observed only for AST (*p* < 0.05; Table 1) but not for albumin or ALP (*p* > 0.05), and a significant interaction between sex and time was not detected for any comprehensive metabolic panel markers. This indicates that temporal changes did not differ significantly between males and females for albumin or ALP. Within‐sex post hoc comparisons revealed significant temporal changes in AST for females only, while no significant time‐related changes were observed in males (Figure 4f,i).

### 3.7 Other tests

There was no significant main effect of sex or interaction between sex and time for prothrombin time (PT) or C‐reactive protein (CRP) (two‐way ANOVA; *p* > 0.05; Table 1). However, males had a larger average increase in PT (2.06 s) compared to females (+0.66 s; *p* = 0.999), while females showed a greater average increase in CRP (6.31 mg/dL vs. 4.11 mg/dL in males; *p* > 0.9999) (Figure 4d,e).

Significant main effects of time were observed for both PT and CRP (Sídák's multiple comparisons test; *p* < 0.05) but without significant interactions, suggesting similar temporal trends across sexes. Within‐sex post hoc comparisons did not reveal statistically significant changes in either marker on the day of diagnosis (Figure 4g,h,j,k).

## 4 DISCUSSION

In this study, we evaluated the differences in vital signs and common blood tests on the day of diagnosis and up to 60 days before and after diagnosis, regardless of existing comorbidities, to investigate the average change in the population. Our previous study demonstrated statistically significant differences in pulse rate and temperature on the day of diagnosis during the 2017–2018 flu season (Martin & Garrett, 2023). This expanded study reveals differences in all vital signs included in this study on the day of diagnosis. Changes in pulse rate (Chow et al., 2021; Nguyen et al., 2014; Williams et al., 2019), respiratory rate (Chow et al., 2021; Nguyen et al., 2014), and temperature (Chow et al., 2021; Nguyen et al., 2014) have been previously associated with influenza infection, while less is discussed about changes in blood pressure (Chow et al., 2021). Although males and females may differ in baseline physiology, our analysis centers on changes in clinically relevant parameters surrounding the time of diagnosis, where identifying robust diagnostic biomarkers is most impactful.

Prior research has demonstrated significant sex‐based differences in the immune response to influenza infection. In both murine models and human studies, females tend to mount stronger innate and adaptive immune responses than males, which may promote faster viral clearance but also increase the risk of immunopathology (Hoffmann et al., 2015; Larcombe et al., 2011; Lorenzo et al., 2011; Robinson, Huber, et al., 2011; Robinson, Lorenzo, et al., 2011). In female mice, influenza A virus (IAV) infection results in heightened pulmonary inflammation and more severe disease outcomes despite comparable viral titers between the sexes (Hoffmann et al., 2015; Larcombe et al., 2011; Lorenzo et al., 2011; Robinson, Huber, et al., 2011; Robinson, Lorenzo, et al., 2011). This immune response is associated with elevated levels of cytokines and chemokines, such as CCL2, CCL3, IFN‐γ, IL‐6, and TNF‐α (Lin et al., 2008; Robinson, Lorenzo, et al., 2011), and is hormonally regulated through sex steroid receptors on immune cells (Biswas et al., 2005; Chadwick et al., 2005; Gilliver, 2010; Klein & Roberts, 2010; McKay & Cidlowski, 1999). These differences in hormonal and immune regulation contribute to sex‐specific outcomes, with females exhibiting greater morbidity and mortality during influenza outbreaks, particularly in pandemic and avian strains (Campbell et al., 2010; Denholm et al., 2010; Eshima et al., 2011; Fielding et al., 2009; Gabriel & Arck, 2014; Klein et al., 2010, 2012; Kumar et al., 2009; Oliveira et al., 2009; World Health Organization, 2008). This study builds on that foundation by identifying clinically detectable, sex‐specific changes in vital signs and blood tests around the time of diagnosis. These findings suggest that tailoring diagnostic strategies to account for sex may improve the identification and management of influenza in clinical settings.

There are additional sex‐specific differences in blood test changes on the day of diagnosis. Female patients exhibited statistically significant differences in 12 blood tests, while males showed significant differences in eight blood tests. Five tests were significantly altered in both sexes: platelets (decreased), calcium (decreased), sodium (decreased), bicarbonate (decreased), and CRP (increased). Ten tests differed by sex: red blood cells (increased in females), white blood cells (decreased in females), hemoglobin (increased in females), glucose (increased in females), potassium (decreased in males), chloride (decreased in females), creatinine (decreased in males), eGFR (increased in males), AST (increased in males), and PT (increased in females). These findings underscore the importance of considering overall and sex‐specific differences when screening patients.

Blood test changes are valuable for assessing disease severity and organ dysfunction during influenza infection (Rendón‐Ramirez et al., 2015). In this study, we observed significant differences in bicarbonate, calcium, chloride, eGFR, and sodium in both sexes, which can help evaluate kidney function and detect potential kidney injury (Gowda et al., 2010). Additionally, males showed significant differences in creatinine and potassium levels. We also observed significant differences in AST in females, which can indicate abnormal liver function. This finding aligns with previous studies, which have shown that abnormal liver function tests are common in hospitalized influenza patients and are associated with worse outcomes (Shafran et al., 2021).

Although CRP data were available for only a small subset of patients (57 patients, 3% of all measured), significant differences were observed in CRP levels across all patients and within both sexes. This supports prior research identifying CRP as a reliable marker for influenza diagnosis (Zhou et al., 2021). Furthermore, they proposed CRP combined with monocyte counts (a component of white blood cells) as a meaningful diagnostic approach (Zhou et al., 2021). Significant differences in CRP and white blood cell counts were observed for females, while in males, only CRP showed statistical significance.

Additionally, there were significant differences in the time required for 14 out of 19 blood tests for both male and female patients over 6 years. There is value in combining changes seen in blood tests with standard viral testing to improve the identification of false positives and false negatives (Ferrari et al., 2020). However, the utility of these tests depends on careful interpretation in the context of a patient's clinical presentation, including diagnostic history and physical examination, to establish appropriate pre‐test probability (Ebell et al., 2012).

Taken together, our findings emphasize the diagnostic value of blood test changes, including both single‐time‐point and time‐dependent differences, in the context of influenza infection. Notably, the pattern of significant tests differed by sex, highlighting the potential benefits of sex‐specific diagnostic strategies. Accounting for these differences may improve the accuracy of influenza diagnosis and support more personalized approaches to patient care.

### 4.1 Limitations and future directions

This study combined Influenza A and Influenza B diagnosed patients and did not include strain‐specific analyses, which were outside the scope of the current investigation. Future studies with detailed strain information should assess whether specific influenza strains differentially impact changes in vital signs and blood tests. Additionally, although this study evaluated average differences across sexes, future work should incorporate other population factors, such as vaccination status, existing comorbidities, age, and BMI, to better understand variability in influenza‐associated laboratory changes across diverse patient groups. Sample sizes varied across clinical parameters, and some groups, such as prothrombin time (PT; 116 patients) and C‐reactive protein (CRP; 57 patients), had small numbers of observations, which may affect the precision or broader applicability of those specific findings. Future studies should also investigate the full dynamics of infection and recovery by examining how physiological values evolve relative to diagnosis, with particular attention to baseline sex differences and temporal trajectories.

## 5 CONCLUSIONS

The addition of blood sample analyses for influenza detection provides more data for influenza surveillance, supporting longitudinal studies, early disease detection, and timely treatment. Here, we highlight the significant physiological changes that occur on the day of influenza diagnosis. Lastly, we emphasize the potential utility of vital signs and blood test changes in clinical assessments and experimental models, such as studies of influenza virus infection in mice.

## FUNDING INFORMATION

No funding information provided.

## ETHICS STATEMENT

Data were extracted from the University of Mississippi Medical Center (UMMC) Patient Cohort Explorer (PCE), which contains de‐identified and date‐shifted clinical information derived from the electronic health record system (EPIC). Because the dataset does not include protected health information (PHI), its use does not constitute human subjects research as defined by 45 CFR 46 and does not require IRB review.

## Supporting information


Figure S1.

Table S1.

Table S2.

## Data Availability

The data analyzed in this study are subject to the following licenses and restrictions: The de‐identified data are accessible to all employees of the University of Mississippi Medical Center. Requests to access these datasets should be directed to cia@umc.edu.
