# Supplementary material for: Sex‐specific changes in vital signs and common blood tests on the day of influenza diagnosis
Source: Physiol Rep. 2025 Aug 7;13(15):e70486. doi: 10.14814/phy2.70486 (PMC12329338; doi:10.14814/phy2.70486)
Supplement: Supplementary file 1 — Figure S1. [file PHY2-13-e70486-s001.pdf]

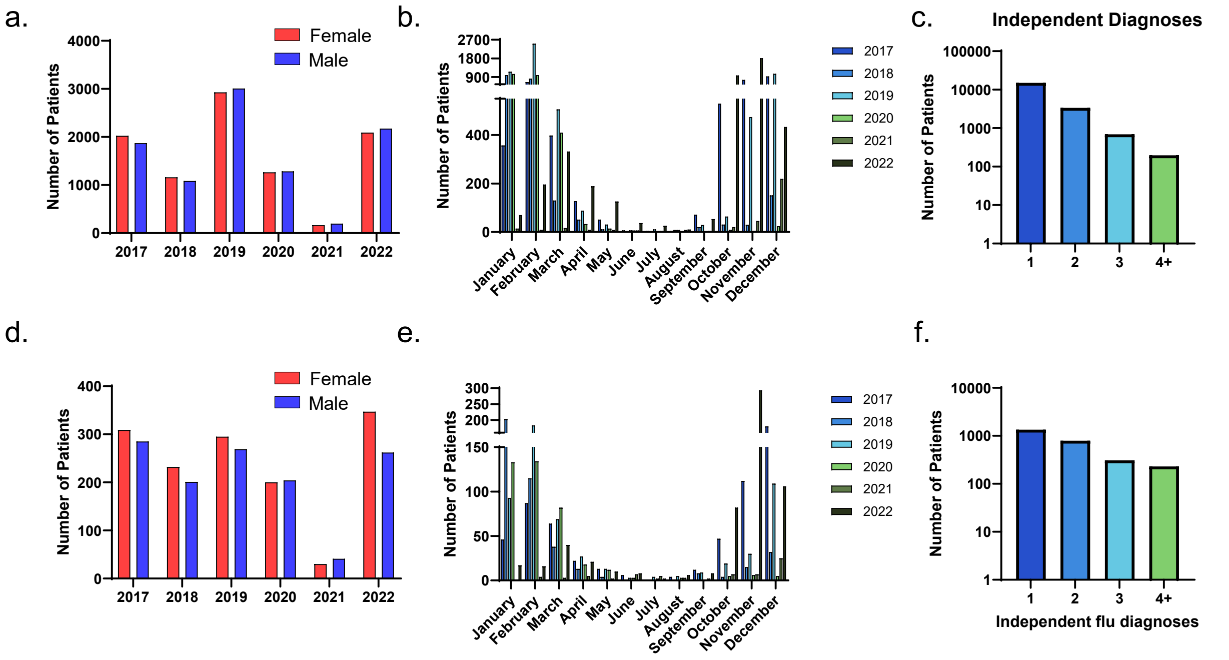


**Supplemental Figure 1.** Demographics of “influenza” diagnosis at encounter and patient level. (a) Total encounters (n = 19,264) for all influenza-diagnosed patients stratified by year, (b) month and year, (c) and number of independent diagnoses. (d) Total encounters (n = 2,675) for influenza-diagnosed patients with measurements stratified by year, (e) month and year (e), and (f) the number of independent diagnoses.
