# Supplementary material for: Sex‐specific changes in vital signs and common blood tests on the day of influenza diagnosis
Source: Physiol Rep. 2025 Aug 7;13(15):e70486. doi: 10.14814/phy2.70486 (PMC12329338; doi:10.14814/phy2.70486)
Supplement: Supplementary file 2 — Table S1. [file PHY2-13-e70486-s003.pdf]

Supplemental Table 1. Counts of influenza-positive patients from January 1, 2017, to December 31, 2022, with available data for each vital sign and blood test parameter. The table includes the total number of diagnosed patients and those with longitudinal data (measurements within 60 days before and after diagnosis), stratified by sex.

	Female		Male	
**Vital Signs**	Influenza diagnosed patients	Patients with longitudinal data	Influenza diagnosed patients	Patients with longitudinal data
Systolic Blood Pressure (mmHg)	7990	382	7539	291
Diastolic Blood Pressure (mmHg)	7990	382	7539	291
Pulse Rate (bpm)	7893	388	7463	294
Respiratory Rate (bpm)	4287	279	3841	208
Temperature (°F)	7147	365	6619	268
**Complete Blood Count**				
Red Blood Cells (M/cmm)	4625	157	4022	134
White Blood Cells (TH/cmm)	4625	390	4022	286
Platelets (TH/cmm)	4625	390	4022	286
Hemoglobin (g/dL)	4728	775	4132	676
Hematocrit (%)	4672	393	4072	287
**Basic Metabolic Panel**				
Calcium (mg/dL)	4222	309	3698	242
Glucose (mg/dL)	6036	310	4634	244
Sodium (mmol/L)	6808	484	5480	414
Potassium (mmol/L)	6809	486	5481	416
Bicarbonate (mmol/L)	6806	482	5480	408
Chloride (mmol/L)	6807	482	5480	408
Blood Urea Nitrogen (BUN) (mg/dL)	6814	484	5483	414
Creatinine (mg/dL)	7155	537	5694	484
Estimated Glomerular Filtration Rate (eGFR) (mL/min/1.73 m2)	3564	500	3040	431
**Comprehensive Metabolic Panel**				
Albumin (g/dL)	3160	274	2712	250
Alkaline Phosphatase (ALP) (U/L)	5505	287	4293	241
Aspartate Aminotransferase (AST) (U/L)	5519	293	4290	240
**Coagulation Panel**				
Prothrombin Time (PT) Test (sec)	1847	48	1511	68
**C-Reactive Protein Test**				
C-Reactive Protein (CRP) (mg/dL)	2225	24	1904	33
