# Supplementary material for: Sex‐specific changes in vital signs and common blood tests on the day of influenza diagnosis
Source: Physiol Rep. 2025 Aug 7;13(15):e70486. doi: 10.14814/phy2.70486 (PMC12329338; doi:10.14814/phy2.70486)
Supplement: Supplementary file 3 — Table S2. [file PHY2-13-e70486-s002.pdf]

Supplemental Table 2. Patient demographics of all influenza-positive patients from January 1, 2017, to December 31, 2022. Data is presented as counts (percentage); categories with less than 20 patients are denoted by <20.

	All Patients		Patients with Measurements	
**Age**	**Female**	**Male**	**Female**	**Male**
<18	6617 (39.01)	7299 (43.03)	352 (18.59)	385 (20.33)
18-29	601 (3.54)	346 (2.04)	100 (5.28)	70 (3.7)
30-39	345 (2.03)	184 (1.08)	85 (4.49)	52 (2.75)
40-49	245 (1.44)	183 (1.08)	73 (3.85)	74 (3.91)
50-64	414 (2.44)	250 (1.47)	194 (10.24)	154 (8.13)
65-74	145 (0.85)	124 (0.73)	101 (5.33)	101 (5.33)
75-84	98 (0.58)	53 (0.31)	67 (3.54)	39 (2.06)
85+	35 (0.21)	24 (0.14)	<20	<20
**BMI**				
≤18	527 (22.01)	612 (25.56)	31 (16.85)	32 (17.39)
19-24	303 (12.66)	273 (11.4)	<20	21 (11.41)
25-29	180 (7.52)	123 (5.14)	24 (13.04)	<20
30-39	165 (6.89)	107 (4.47)	<20	<20
40+	80 (3.34)	24 (1)	<20	<20
**First Race**				
American Indian or Alaska Native	<20	20 (0.12)	<20	<20
Asian	71 (0.42)	73 (0.43)	<20	<20
Black or African American	5542 (32.67)	5282 (31.14)	693 (36.59)	578 (30.52)
Hispanic	40 (0.24)	40 (0.24)	<20	<20
MS Band Choctaw Indian	<20	<20	<20	<20
Multiracial	90 (0.53)	90 (0.53)	<20	<20
Native Hawaiian or Other Pacific Islander	<20	<20	<20	<20
Other or Unknown	451 (2.66)	445 (2.62)	31 (1.64)	<20
White or Caucasian	2281 (13.45)	2497 (14.72)	265 (13.99)	281 (14.84)
